# Editorial: Physio-logging in humans: recent advances and limitations in wearable devices for biomedical applications

**DOI:** 10.3389/fphys.2025.1639428

**Published:** 2025-06-25

**Authors:** Céderick Landry, Mohammad Yavarimanesh, Colin K. Drummond

**Affiliations:** ^1^ Mechanical Engineering Department, Université de Sherbrooke, Sherbrooke, QC, Canada; ^2^ Applied Data Science, University of San Diego, San Diego, CA, United States; ^3^ Biomedical Engineering, Case Western Reserve University, Cleveland, OH, United States

**Keywords:** biomedical sensors, wearable sensors, human performance, physical sensors, chemical sensors

The recent advancements in wearables and machine learning have paved the way for unparalleled approaches to monitor physiological parameters, prevent diseases and medical conditions, and to assist, and treat patients that suffer from them. These approaches also show great potential in studying human physiology in extreme conditions. Wearable devices can provide real-time information about human health and wellbeing in extreme environments, enabling early detection of any changes or abnormalities in normal physiological function. In addition, wearables and recent advances in physio-logging can alleviate the impact of numerous diseases, and medical conditions globally. These approaches will impact our life also by reducing the cost of healthcare and increasing patients’ quality of life. Noteworthy strides have already been accomplished, evoking enthusiasm among patients and researchers alike.

Based on the considerations outlined above, this Research Topic, entitled “Physio-logging in Humans: Recent Advances and Limitations in Wearable Devices for Biomedical Applications,” aimed to showcase original multidisciplinary research focused on the development, validation, translational Research Topic and practical application of wearable technologies for physiological monitoring. Following rigorous peer review, eight original research papers were selected for inclusion in this Research Topic.

The work of Brady et al. aimed to explore foundational capabilities and feasibility of wearable physio-logging for remote monitoring. Recent advances in wearable technologies have expanded the scope of physio-logging in biomedical applications. Studies demonstrate the feasibility of remote health monitoring using apps like Labs Without Walls and devices such as the Apple Watch, with high adherence across diverse age groups. In a clinical trial conducted over 8 weeks, participants provided high-quality passive and active data with strong adherence and usability. The findings support wearable-based physio-logging as a scalable, user-friendly approach for decentralized health monitoring, highlighting its potential for broad population studies and future enhancements through gamification and improved survey design.

Understanding the validity of wearable sensors to measure specific metrics play a crucial role for clinical adoption. In this line of work, Icenhower et al. conducted validation studies showing PPG-based heart rate measurements are largely unaffected by skin tone, however, accuracy declined during rapid activity changes compared to ECG readings. These results support the reliability of PPG across diverse populations, while highlighting the need for continued validation of wearable devices under dynamic conditions to ensure equitable and accurate physio-logging in biomedical applications.

With a focus on translating technology to market for low-resource settings, Mendt et al. assessed consumer-grade wearables against research-grade devices during physical activity. While the consumer grade device performed well for heart rate at low intensity, its accuracy declined with exertion. Step count, energy expenditure, and temperature readings also showed limited reliability. These findings, again, highlight both the potential and limitations of consumer wearables for physio-logging, suggesting they may be useful for long-term monitoring in low-resource settings, but are not yet suitable for precise clinical applications or, at least, should be validated according to their intended use-case.

In the spirit of interdisciplinary research combining biomedical tech and immersive media, the human factors research of Medarević et al. assessed the ability of two wearable devices—the Empatica E4 and Faros 360—to detect physiological distress in interactive virtual reality (VR) environments. Using heart rate metrics, both devices successfully identified distress, particularly during interactive VR scenes. The Faros 360 showed superior signal quality and consistency, though both devices demonstrated good agreement in heart rate measurement. These findings highlight the potential of wearable physio-logging tools in immersive settings, supporting their use in adaptive VR therapies and user experience optimization. The study also underscores the importance of device-specific performance in accurately capturing emotional and physiological states.

Practical application of wearables must also anticipate use in extreme environments Pernett et al. concluded that a custom-made chest strap equipped with strain gages, similar to consumer-grade devices, still faces limitations in accuracy under physical stress. Specifically, the study evaluated the ability of their force sensor to estimate hyperventilation risk in freedivers by predicting end-tidal CO_2_ levels. Data from 21 athletes showed that chest movement amplitude and respiratory rate could explain 34% of CO_2_ variability, suggesting potential for detecting unintentional hyperventilation—a key blackout risk. These findings highlight the promise of wearable physio-logging for enhancing safety in high-risk sports, while underscoring the need for further validation and improved algorithms for freediving safety.

In a dynamic field such as wearables, technical innovations and modeling abound, Ogata et al. developed a personalized method for estimating energy expenditure during heavy physical labor using wearable accelerometers and heart rate sensors. By calibrating individual models, the combined approach significantly outperformed accelerometer-only estimates. These findings underscore the limitations of single-sensor systems and highlight the value of multimodal wearables in extreme environments. These results may lead to improved health and nutrition planning for disaster relief teams and advancing physio-logging applications in real-world, high-stress scenarios.

Machine learning is increasingly featured in wearables research and Kishor Kumar Reddy et al. introduces a ResNet-LSTM deep learning model for non-invasive blood pressure estimation using ECG and PPG signals. Designed for Smart Health Monitoring in remote or underserved areas, the model achieved high accuracy despite greater computational demands. Its strong performance across datasets highlights the potential of AI-powered wearable physio-logging to enhance real-time cardiovascular monitoring and address limitations of traditional cuff-base blood pressure measurement. In a similar way, Dervieux et al. aimed to explore predictions and limitations of models for non-heated transcutaneous CO_2_ sensors that have the potential to allow more accessible, real-time monitoring of arterial CO2 partial pressure in clinical and remote settings to monitor patients severe respiratory disorders. This study examined how skin temperature influences transcutaneous CO_2_ diffusion, a key factor in wearable capnometer performance. In 40 adults, skin conductivity, CO_2_ exhalation, and blood flow increased significantly at 35–38°C—temperatures achievable without active heating. These findings support the feasibility of non-heated tcpCO_2_ sensors, advancing wearable physio-logging by addressing a major limitation in current monitors and enabling more practical, continuous respiratory tracking in both clinical and remote settings.

Collectively, these contributions underscore the growing reliability, versatility, and challenges of wearable physio-logging, paving the way for more inclusive, adaptive, and scalable health technologies.

